# Profluorescent substrates for the screening of olefin metathesis catalysts

**DOI:** 10.3762/bjoc.11.203

**Published:** 2015-10-12

**Authors:** Raphael Reuter, Thomas R Ward

**Affiliations:** 1 Department of Chemistry, University of Basel, Spitalstrasse 51, CH-4056 Basel, Switzerland

**Keywords:** fluorescence, microplate screening, ring closing metathesis

## Abstract

Herein we report on a 96-well plate assay based on the fluorescence resulting from the ring-closing metathesis of two profluorophoric substrates. To demonstrate the validity of the approach, four commercially available ruthenium-metathesis catalysts were evaluated in six different solvents. The results from the fluorescent assay agree well with HPLC conversions, validating the usefulness of the approach.

## Introduction

Since its discovery in the 1950s, olefin metathesis has developed into one of the most powerful catalytic reactions both in research as well as in industrial applications [1–3]. This is mostly due to its excellent chemoselectivity, tolerance of many functional groups and its atom economy [4]. Chemists treasure its extraordinary versatility. From the production of polymers [5–6] and petrochemicals to the synthesis of complex natural products [7], olefin metathesis has been established as a useful tool for solving numerous synthetic challenges. In more recent applications, metathesis has also been used in chemical biology, either in the form of an artificial metalloenzyme [8–10] or for the post-translational modification of proteins [11]. To address these various challenges, a vast number of carbene complexes based on different transition metals have been prepared and tailored towards specific applications [12]. With the ultimate aim of identifying new olefin metathesis catalysts using high-throughput screening, we set out to develop and evaluate olefinic substrates amenable to a 96-well plate screening format.

## Results and Discussion

A quick and highly sensitive analytical method that is suitable for the fast detection and quantification of small quantities of a product is fluorescence spectroscopy. In particular, biological applications heavily rely on fluorescence-based visualization techniques [13]. For this purpose, a large variety of fluorescent probes have been developed that react to different chemical stimuli [14]. Although previous work on the development of fluorescent olefin metathesis catalysts [15–16] exists, to our knowledge, the concept of fluorescent probes based on ring-closing metathesis is new and could be of value to chemical biologists. Since microplates are a very common and practical tool for biological applications, we developed a screening assay in 96-well plate format to quickly evaluate the reaction kinetics of different commercially available metathesis catalysts. Since fluorescence spectroscopy is a highly sensitive technique, we aimed at using a low catalyst concentration (e.g., 100 µM) in a small reaction volume (150 µL). With this format, only 1 mg of catalyst is required to perform fifty to a hundred kinetic experiments.

**Figure 1:**
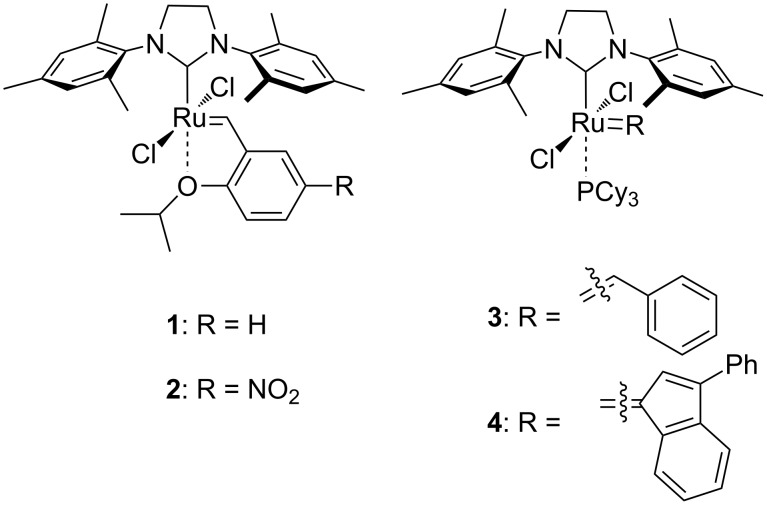
Catalysts **1–4** tested for the metathesis of profluorescent substrates.

For this proof-of-principle study, we selected four commercially available, second generation-type catalysts **1–4** (Figure 1). These catalysts were mainly chosen because of their high stability towards both air and moisture. Catalysts **1** and **2** are the phosphine-free Grubbs–Hoveyda and Grela-type catalysts bearing different isopropoxystyrene ligands. Catalysts **3** and **4** are phosphine-containing Grubbs-type catalysts with either a benzylidene ligand or an indenylidene ligand.

**Scheme 1:**
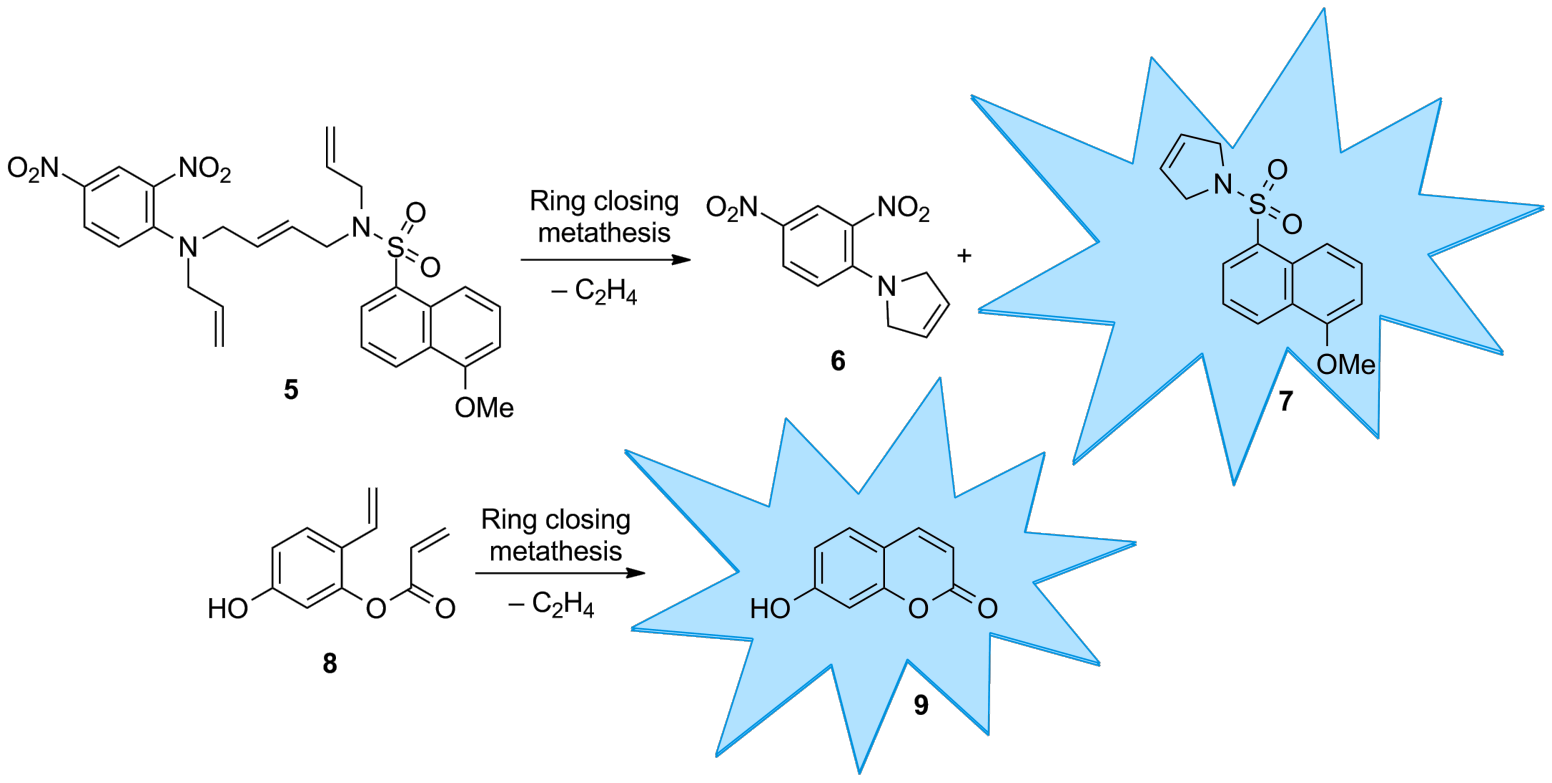
Two profluorescent substrates yielding fluorescent products upon ring-closing metathesis.

As a model reaction, we selected ring-closing metathesis and developed two profluorescent substrates that yield a fluorescent product upon ring-closing metathesis (Scheme 1). Substrate **5** consists of a fluorescent 5-methoxynaphthalene-1-sulfonamide moiety that is connected by an internal double bond to a 2,4-dinitroaniline core, acting as a fluorescence quencher [17]. Both the sulfonamide of the fluorophore and the aniline group of the quencher bear another allyl group. Upon relay ring-closing metathesis, the fluorophore and quencher are disconnected resulting in the fluorescent product **7**. A similar linker concept has previously been implemented for a solid-phase linker in the synthesis of oligosaccharides [18–19]. The second profluorescent molecule selected was diolefin **8**, which yields fluorescent 7-hydroxycoumarin (umbelliferone) (**9**) upon ring-closing metathesis. The synthesis of coumarin derivatives using this approach was described in previous publications [20–21]. By introducing an electron donor in the 7-position, a fluorescent product is obtained upon ring-closing metathesis [22].

### Synthesis of the profluorescent substrates

**Scheme 2:**
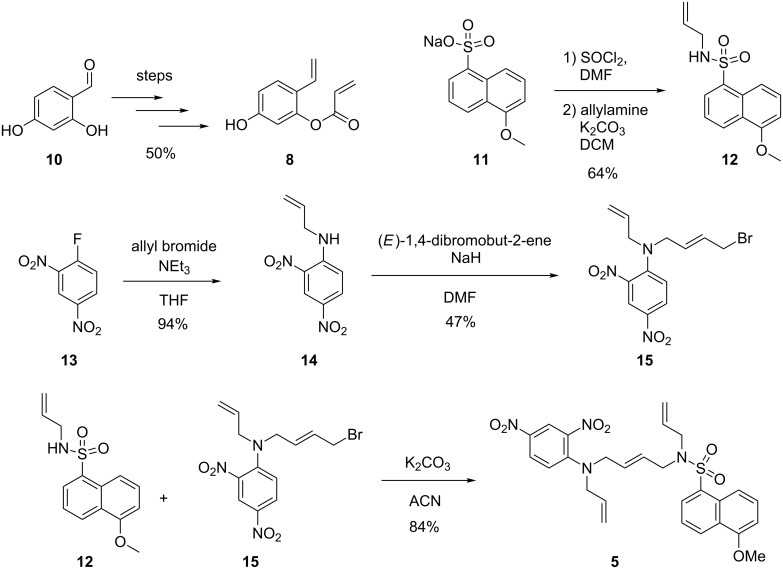
Synthesis of the two profluorescent substrates amenable to ring-closing metathesis.

The synthesis of profluorescent substrate **8** leading to umbelliferone after ring-closing metathesis was carried out according to a published, four-step procedure starting from 2,4-dihydroxybenzaldehyde (**10**) with an overall yield of 50% [23–24]. The synthesis of the fluorophore–quencher substrate **5** was achieved relying on two converging synthons (Scheme 2). The fluorophore part of the molecule was synthesized starting from sodium 5-methoxynaphthalene-1-sulfonate (**11**), which was prepared according to a known procedure [25]. It was then transformed to the corresponding allyl sulfonamide **12** by reacting the corresponding acid chloride with allylamine. The quencher part of the molecule was prepared from commercial 1-fluoro-2,4-dinitrobenzene (**13**). Following an alkylation step with allyl bromide, it was reacted with an excess of (*E*)-1,4-dibromobut-2-ene to selectively afford the mono alkylated product. A strong base (e.g., NaH) was crucial to achieve complete deprotonation of the highly deactivated aniline [26]. The fluorophore and quencher parts were finally connected in high yield relying on a nucleophilic substitution.

### Ring-closing metathesis of substrates

**Figure 2:**
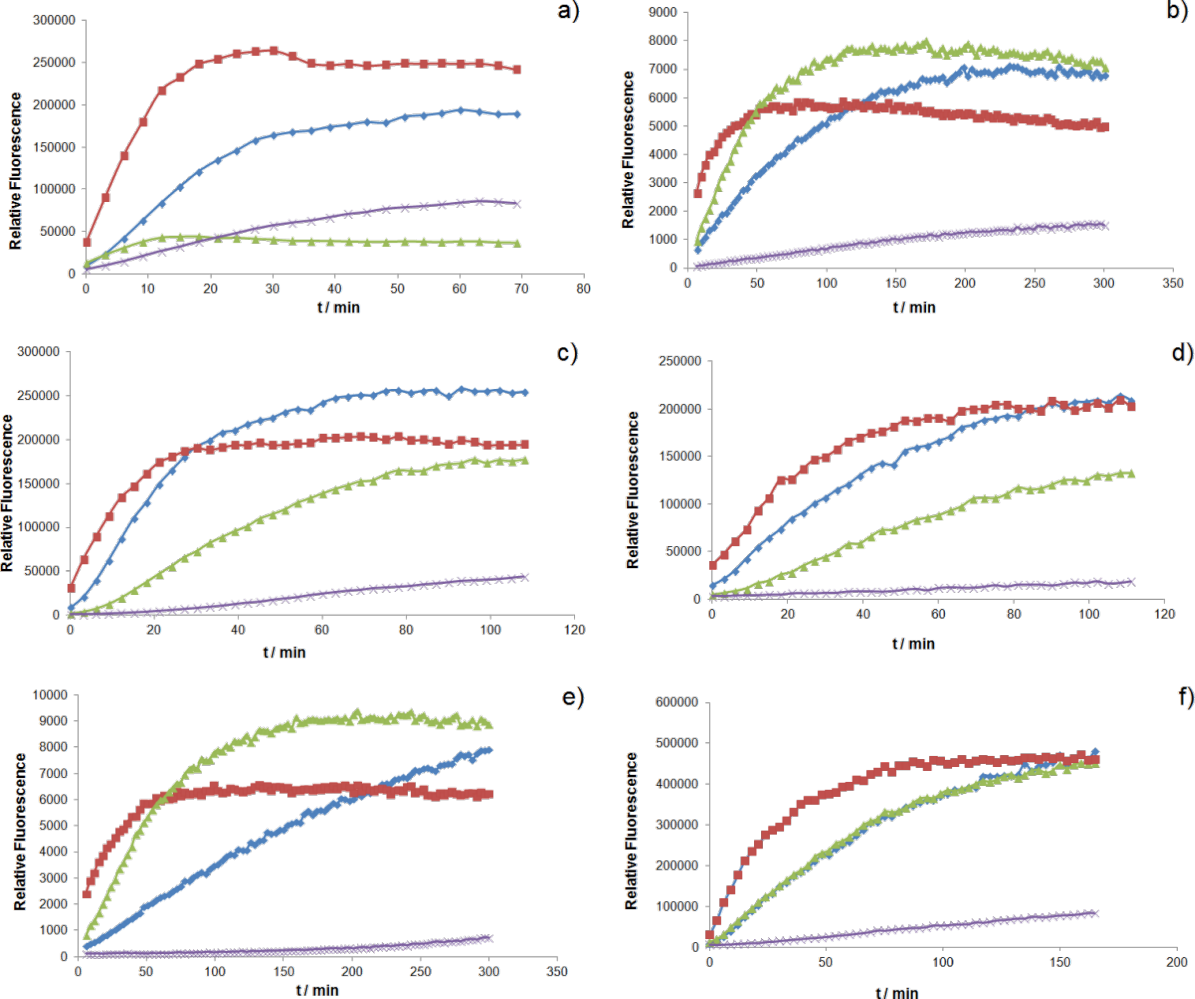
Fluorescence evolution resulting from closing metathesis of umbelliferone precursor **8** (λ_excitation_ = 350 nm and λ_emission_ = 380 nm). Different catalysts **1**–**4** (**1**: blue; **2**: red; **3**: green; **4**: purple) were screened in different solvents (a: acetic acid; b: 2-methylpentanone; c: toluene; d: *o*-xylene; e: γ-butyrolactone; f: anisole).

With the aim of miniaturizing and automatizing the screening effort for the identification of ring-closing metathesis catalysts, a 96-well plate format screening was developed, relying on a total volume of 150 µL per experiment and 100 µM catalyst in the presence of 10 mM substrate. To demonstrate the versatility of the method, the kinetics of the ring-closing metathesis with umbelliferone precursor **8** were determined with different catalysts **1–4** (Figure 2). It is known that acryloyl ester substrates are poor substrates which typically give low yields with 1^st^ generation precatalysts [27]. As the reactions were performed in air and with very small volumes, high boiling solvents with different structural features were selected. From the screening, the following features were apparent:

1. Grubbs–Hoveyda and Grela catalysts **1** and **2** perform better for substrate **8** in most solvents.
2. For solvents that contain a carbonyl or carboxyl function (i.e., 2-methylpentanone and γ-butyrolactone), the Grubbs 2^nd^ generation catalyst **3** performs best.
3. In all cases, the initial ring-closing metathesis rates are highest with precatalyst **2**.

**Figure 3:**
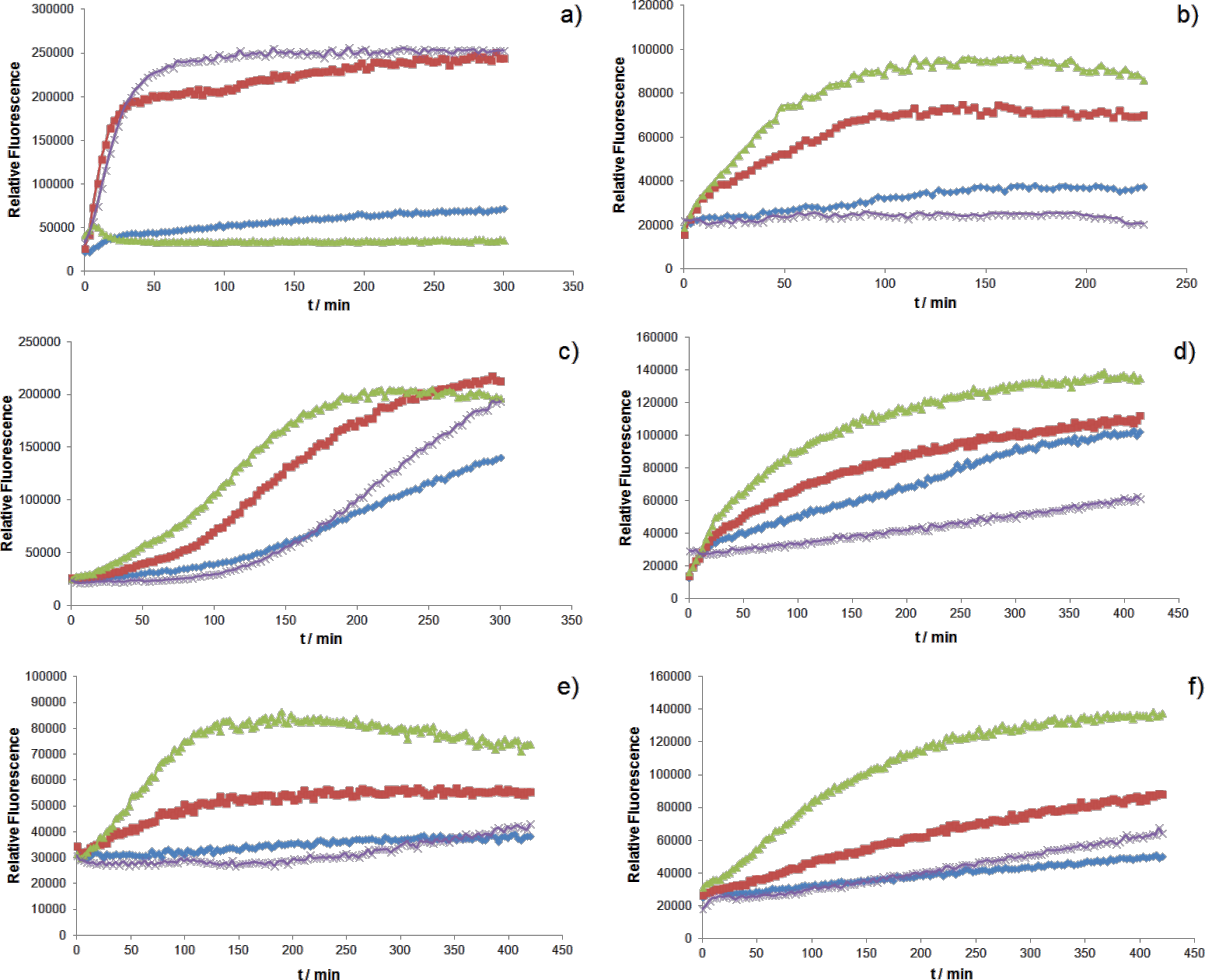
Fluorescence evolution resulting from closing metathesis of fluorescence–quencher substrate **5** (λ_excitation_ = 320 nm and λ_emission_ = 400 nm). Different catalysts **1**–**4** (**1**: blue; **2**: red, **3**: green, **4**: purple) were screened in different solvents (a: acetic acid; b: 2-methylpentanone; c: toluene; d: *o*-xylene; e: γ-butyrolactone; f: anisole).

The same experiments were conducted with the fluorophore–quencher substrate **5** (Figure 3). In this case, the concentration of the substrate was reduced to 1 mM to miminize intermolecular quenching of fluorophore **7** by dinitroaniline **6**. The following observations can be made:

1. Generally, a higher activity is observed for the Grubbs 2^nd^ generation catalyst **3** as compared to the umbelliferone substrate **8**. The only exception is when acetic acid is used as the solvent.
2. The catalyst **4**, being nearly inactive for the electron-poor substrate **8**, showed increased activity for substrate **5**, especially in acetic acid.
3. Surprisingly, catalyst **1** was (one of) the worst performing for this bulky substrate.

**Figure 4:**
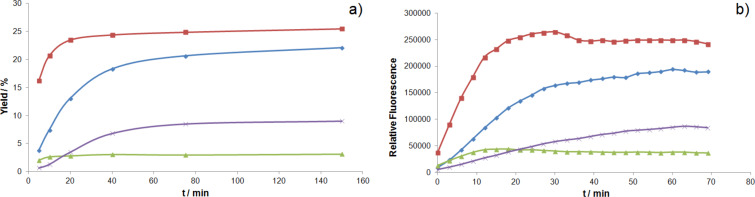
Comparison of kinetics measured by HPLC a) and by a plate reader b) for the ring-closing metathesis of **8** in acetic acid.

To validate the kinetics determined by fluorescence, the reaction progress was monitored by HPLC for umbelliferone precursor **8** in acetic acid (Figure 4). Gratifyingly, both the fluorescence and HPLC techniques result in very similar trends.

## Conclusion

In summary, two profluorescent substrates, **5** and **8**, were prepared and fully characterized. Upon ring-closing metathesis, they produce a fluorescent signal thus allowing a straightforward screening in a 96-well plate format. The validity of the approach was demonstrated by screening four commercially available catalysts **1**–**4** in different solvents. Comparison between the kinetics determined by HPLC and fluorescence showed good agreement. These profluorescent substrates could be prototypes for more complex structures that could find applications in ring-closing metathesis for biological applications by fluorescence microscopy.

## Experimental

**General Methods:** The ^1^H and ^13^C NMR spectra were recorded on Bruker 400 MHz and 500 MHz spectrometers. The chemical shifts are reported in ppm (parts per million). Electrospray ionization mass spectra (ESIMS) were recorded on a Bruker FTMS 4.7T bioAPEX II spectrometer. HRMS was measured on a Bruker maXis 4G QTOF-ESI spectrometer. HPLC was conducted on a Waters Acquity H-Class Bio UPLC device, using a BEH C-18 reversed-phase column. Starting materials and reagents were purchased from commercial sources and used without further purification. HPLC grade solvents were used if not mentioned otherwise. For the fluorescence measurements, a TECAN Infinite M1000 platereader was used.

***N*-Allyl-5-methoxynaphthalene-1-sulfonamide (12).** To a DMF solution (30 mL) of sodium 5-methoxynaphthalene-1-sulfonate (**11**, 1.30 g, 5.00 mmol, 1.00 equiv), cooled on an ice bath, thionyl chloride (1.09 mL, 15.0 mmol, 3.00 equiv) was added dropwise. After the complete addition, the ice bath was removed and the reaction was stirred at rt for 3 h. Then, it was poured onto 300 mL of ice water and extracted with ethyl acetate (3 × 100 mL). The combined extracts were dried over MgSO_4_ and the solvent was removed at reduced pressure. The residual oil was taken up into DCM (100 mL). Allylamine was slowly added to the solution, while stirring. After complete addition, the reaction mixture was allowed to stir overnight. The solvent was removed under vacuum and the residue was purified by flash column chromatography (silica gel, cyclohexane/EtOAc 1:1) to obtain a colourless solid (893 mg, 64%). ^1^H NMR (400 MHz, CDCl_3_) δ 8.46 (d, ^3^*J*_HH_ = 8.4 Hz, 1H), 8.22 (d, ^3^*J*_HH_ = 8.7 Hz, 1H), 8.15 (s, 1H), 8.14 (d, ^3^*J*_HH_ = 7.3 Hz, 1H), 7.66–7.58 (m, 2H), 7.13 (d, ^3^*J*_HH_ = 7.6 Hz, 1H), 5.56 (ddt, ^3^*J*_HH_ = 17.1 Hz, ^3^*J*_HH_ = 10.3 Hz, ^3^*J*_HH_ = 5.5 Hz, 1H), 5.05 (ddt, ^3^*J*_HH_ = 17.1 Hz, ^2^*J*_HH_ = 1.7 Hz, ^4^*J*_HH_ = 1.7 Hz, 1H), 4.91 (ddt, ^3^*J*_HH_ = 10.3 Hz, ^2^*J*_HH_ = 1.5 Hz, ^4^*J*_HH_ = 1.5 Hz, 1H), 4.01 (s, 3H), 3.45 (d, ^3^*J*_HH_ = 5.5 Hz); ^13^C NMR (100 MHz, CDCl_3_) δ 155.1, 135.6, 134.2, 128.8, 128.6, 128.3, 126.9, 125.7, 123.8, 116.8, 116.2, 105.4, 55.9, 44.8; HRMS [ESI(+)–TOF] *m*/*z*: [M + H]^+^ calcd for C_14_H_16_NO_3_S, 278.0851; found, 278.0845; Elemental analysis: anal. calcd for C_14_H_15_NO_3_S: C, 60.63; H, 5.45; N, 5.05; found: C, 60.48; H, 5.58; N, 5.25.

***N*-Allyl-2,4-dinitroaniline (14).** A flask was charged with 2,4-dinitrofluorobenzene (**13**, 854 mg, 4.59 mmol, 1.00 equiv) and THF (10 mL). First, triethylamine (710 µL, 5.05 mmol, 1.10 equiv) and then allylamine (378 µL, 5.05 mmol, 1.10 equiv) was added and the mixture was stirred for 2 h at rt until the TLC showed complete consumption. The solvent was removed under reduced pressure and the residue was purified by flash chromatography (silica gel, cyclohexane/EtOAc 3:1) to obtain 960 mg of a yellow solid (94%). ^1^H NMR (400 MHz, CDCl_3_) δ 9.15 (d, ^4^*J*_HH_ = 2.7 Hz, 1H), 8.69 (bs, 1H), 8.27 (dd, ^3^*J*_HH_ = 9.5 Hz, ^4^*J*_HH_ = 2.7 Hz, 1H), 6.92 (d, ^3^*J*_HH_ = 9.5 Hz, 1H), 5.96 (ddt, ^3^*J*_HH_ = 17.3 Hz, ^3^*J*_HH_ = 10.2 Hz, ^3^*J*_HH_ = 5.1 Hz, 1H), 5.39–5.30 (m, 1H), 4.13–4.08 (m, 1H); ^13^C NMR (100 MHz, CDCl_3_) δ 148.3, 136.4, 131.6, 130.6, 130.3, 124.2, 118.3, 114.3, 45.7; HRMS [ESI(+)–TOF] *m*/*z*: [M + H]^+^calcd for C_9_H_10_N_3_O_4_, 224.0671; found, 224.0663. Elemental analysis: anal. calcd for C_9_H_9_N_3_O_4_: C, 48.43; H, 4.06; N, 18.83; found: C, 48.26; H, 4.15; N, 19.08.

**(*E*)-*N*-Allyl-*N*-(4-bromobut-2-en-1-yl)-2,4-dinitroaniline (15).** To a solution of *N*-allyl-2,4-dinitroaniline (**14**, 400 mg, 1.79 mmol, 1.00 equiv) in dry DMF (7 mL), NaH (60% in mineral oil, 143 mg, 3.58 mmol, 2.00 equiv) and (*E*)-1,4-dibromobut-2-ene (1.53 g, 7.16 mmol, 4.00 equiv) were added. The mixture was stirred at 70 °C for 16 h. The mixture was diluted with EtOAc (50 mL), washed with water (2 × 50 mL) and dried over MgSO_4_. The solvent was removed under vacuum and the residue was purified by flash column chromatography (silica gel, cyclohexane/EtOAc 5:1) to obtain 300 mg of red oil (47%). ^1^H NMR (400 MHz, CDCl_3_) δ 8.66 (d, ^4^*J*_HH_ = 2.7 Hz, 1H), 8.21 (dd, ^3^*J*_HH_ = 9.4 Hz, ^4^*J*_HH_ = 2.7 Hz, 1H), 7.09 (d, ^3^*J*_HH_ = 9.4 Hz, 1H), 5.99–5.87 (m, 1H), 5.86–5.70 (m, 2H), 5.33 (d, ^3^*J*_HH_ = 10.3 Hz, 1H), 5.28 (d, ^3^*J*_HH_ = 17.2 Hz, 1H), 3.86–3.90 (m, 6H); ^13^C NMR (100 MHz, CDCl_3_) δ 148.1, 137.9, 137.7, 131.8, 131.3, 128.4, 127.7, 123.7, 120.1, 119.4, 54.6, 53.2, 31.0; HRMS [ESI(+)–TOF] *m*/*z*: [M + H]^+^ calcd for C_13_H_15_BrN_3_O_4_, 356.0246; found, 356.0239.

**Substrate 5.** A round bottom flask was charged with *N*-allyl-5-methoxynaphthalene-1-sulfonamide (**12**, 449 mg, 1.62 mmol, 1.00 equiv), (*E*)-*N*-allyl-*N*-(4-bromobut-2-en-1-yl)-2,4-dinitroaniline (**15**, 610 mg, 1.71 mmol, 1.06 equiv), K_2_CO_3_ (672 mg, 4.86 mmol, 3.00 equiv) and acetonitrile (20 mL). The mixture was stirred at 70 °C overnight and filtered. The filtrate was concentrated at reduced pressure and the residue was purified by flash column chromatography (silica gel, cyclohexane/EtOAc 3:1) to obtain 750 mg of an orange semi-solid (84%). ^1^H NMR (400 MHz, CDCl_3_) δ 8.60 (d, ^4^*J*_HH_ = 2.7 Hz, 1H), 8.52 (d, ^3^*J*_HH_ = 8.4 Hz, 1H), 8.26 (dd, ^3^*J*_HH_ = 7.4 Hz, ^4^*J*_HH_ = 1.3 Hz, 1H), 8.14–8.08 (m, 2H), 7.55–7.46 (m, 2H), 6.94–6.86 (m, 2H), 5.72–5.34 (m, 4H), 5.26 (d, ^3^*J*_HH_ = 10.3 Hz, 1H), 5.17 (d, ^3^*J*_HH_ = 17.1 Hz, 1H), 5.14–5.05 (m, 2H), 4.03 (s, 3H), 3.86 (dd, ^3^*J*_HH_ = 17.7 Hz, ^3^*J*_HH_ = 6.1 Hz, 4H), 3.74 (dd, ^3^*J*_HH_ = 17.8 Hz, ^3^*J*_HH_ = 5.3 Hz, 4H); ^13^C NMR (100 MHz, CDCl_3_) δ 155.8, 147.9, 137.6, 137.4, 134.3, 132.7, 131.4, 130.8, 129.9, 129.5, 128.41, 128.39, 127.61, 127.55, 126.7, 123.6, 123.3, 120.0, 119.3, 119.1, 116.8, 104.8, 55.7, 54.5, 53.2, 49.1, 47.1; HRMS [ESI(+)–TOF]: [M + H]^+^ calcd for C_27_H_29_N_4_O_7_S, 553.1757; found, 553.1756; Elemental analysis: anal. calcd for C_27_H_28_N_4_O_7_S: C, 58.69; H, 5.11; N, 10.14; found: C, 58.83; H, 5.45; N, 10.15.

**Ring-closing metathesis in 96-well plates:** To each well, 142 µL of the selected solvent (cooled at 5 °C to minimize evaporation) was pipetted. Then, 5 µL of a 3 mM precatalyst stock solution was added. Finally, 3 µL of the substrate stock solution was added (for the coumarin substrate **8** a 1 M stock solution was used, for the fluorophore quencher substrate **5**, a 50 mM stock solution was used). The plate was then sealed with transparent plastic foil and analyzed using a fluorescence plate reader at 27 °C, in 3 min measuring intervals with shaking between the measurements (substrate **5**: λ_excitation_ = 320 nm; λ_emission_ = 400 nm; substrate **8**: λ_excitation_ = 350 nm; λ_emission_ = 380 nm).

## Supporting Information

File 1 NMR spectra of synthesized compounds.
