# Supplementary material for: Profluorescent substrates for the screening of olefin metathesis catalysts
Source: Beilstein J Org Chem. 2015 Oct 12;11:1886–92. doi: 10.3762/bjoc.11.203 (PMC4660973; doi:10.3762/bjoc.11.203)
Supplement: File 1 — NMR spectra of synthesized compounds. [file Beilstein_J_Org_Chem-11-1886-s001.pdf]

Supporting Information

for

Profluorescent substrates for the screening of olefin metathesis catalysts

Raphael Reuter and Thomas R. Ward*

Address: Department of Chemistry, University of Basel, Spitalstrasse 51, CH-4056 Basel, Switzerland

Email: Thomas R. Ward - thomas.ward@unibas.ch

* Corresponding author

NMR spectra of synthesized compounds

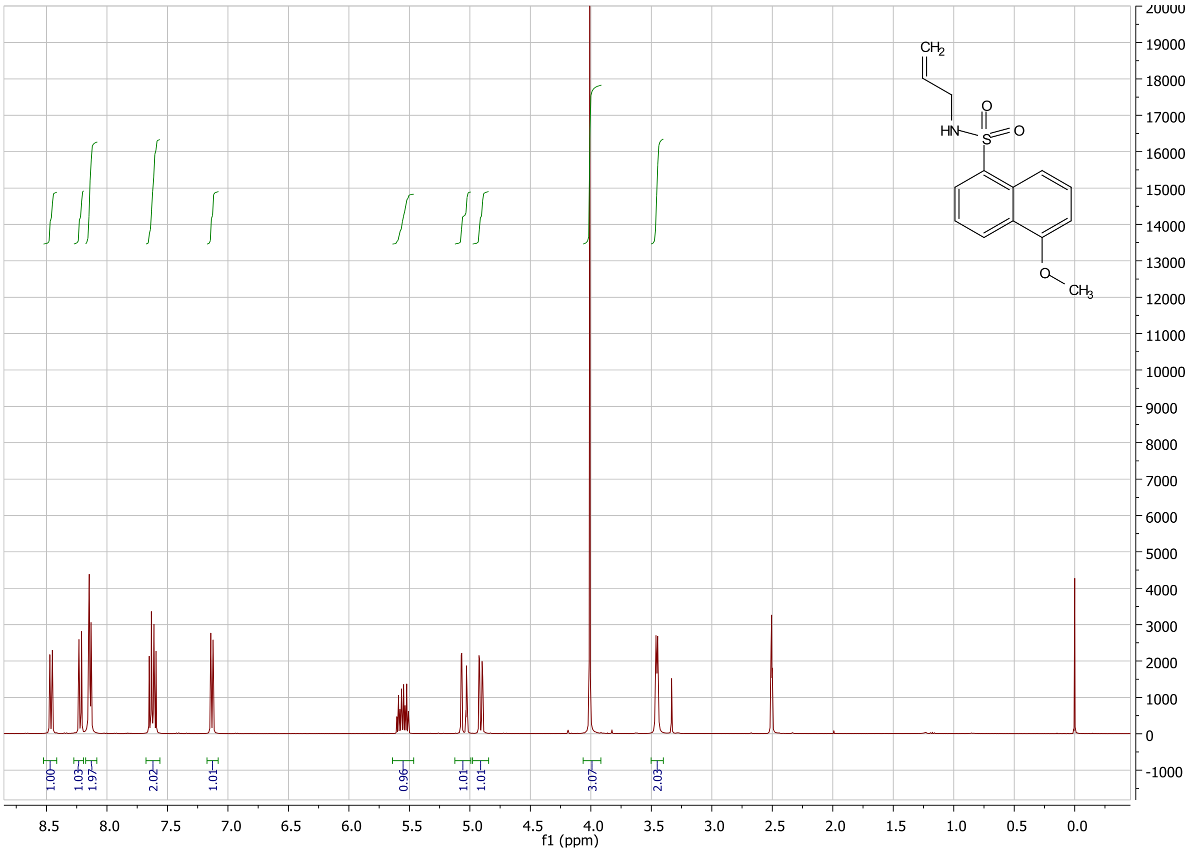
CH2
HN
S
O
O
O
CH3
1.00
1.03
1.97
2.02
1.01
0.96
1.01
1.01
3.07
2.03
f1 (ppm)

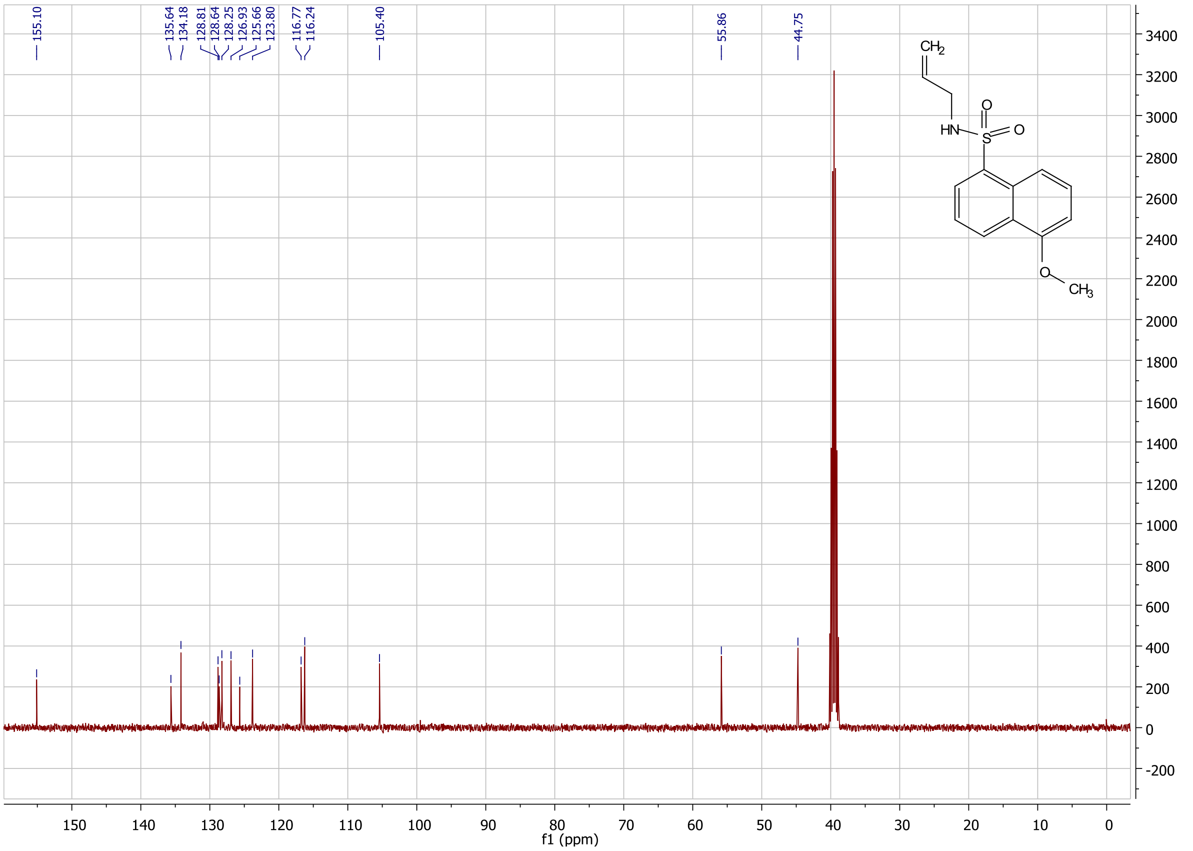
155.10
135.64
134.18
128.81
128.64
128.25
126.93
125.66
123.80
116.77
116.24
105.40
55.86
44.75
CH2
HN
S
O
O
O
CH3
f1 (ppm)

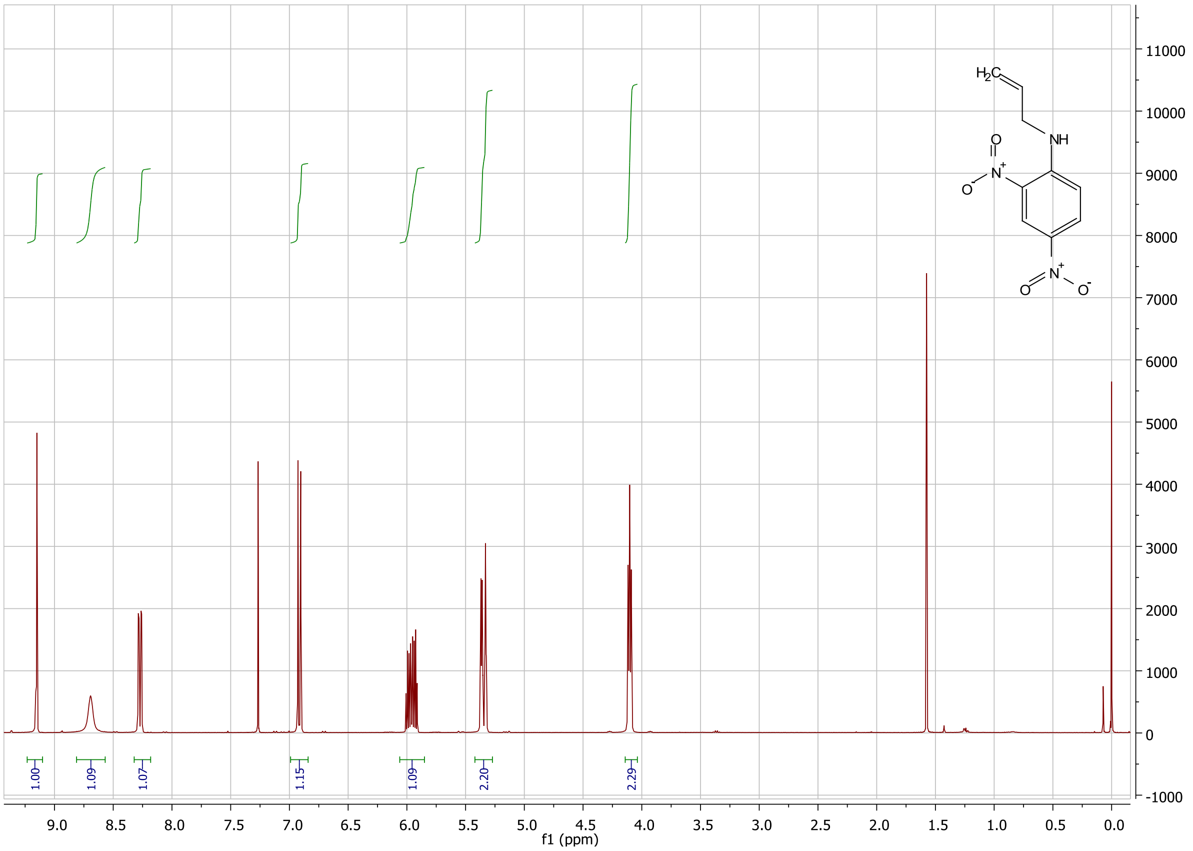

1.00
1.09
1.07
1.15
1.09
2.20
2.29
f1 (ppm)

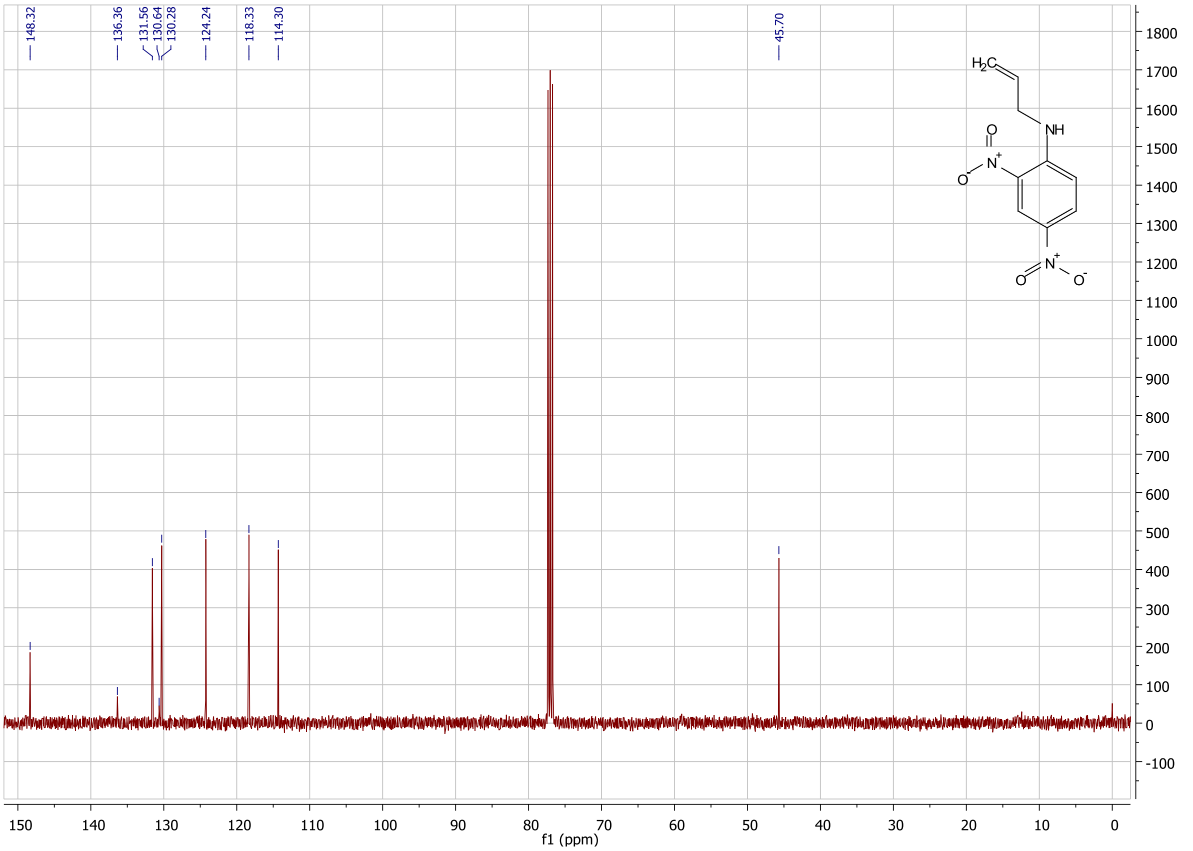

148.32
136.36
131.56
130.64
130.28
124.24
118.33
114.30
45.70
f1 (ppm)

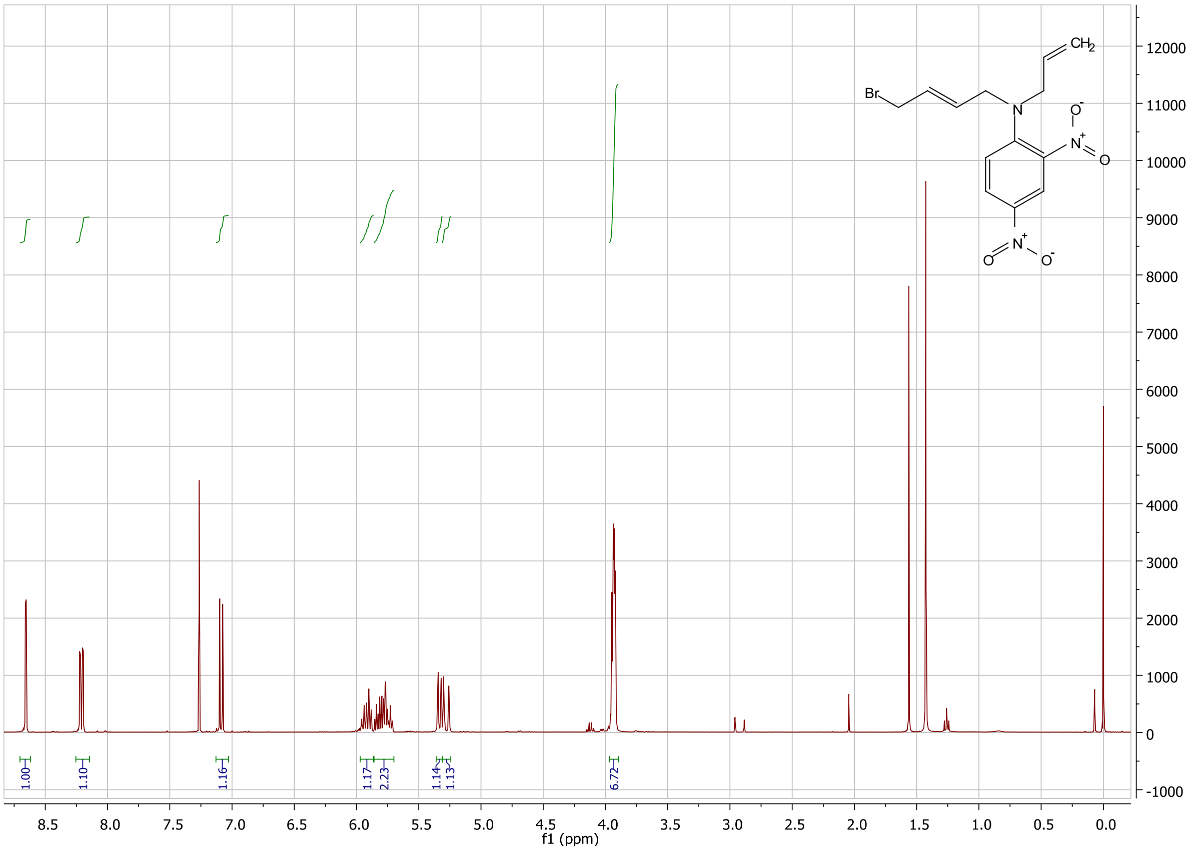

CH2
Br
N
O-
N+
O
N+
O
O-
1.00
1.10
1.16
1.17
2.23
1.14
1.13
6.72
f1 (ppm)

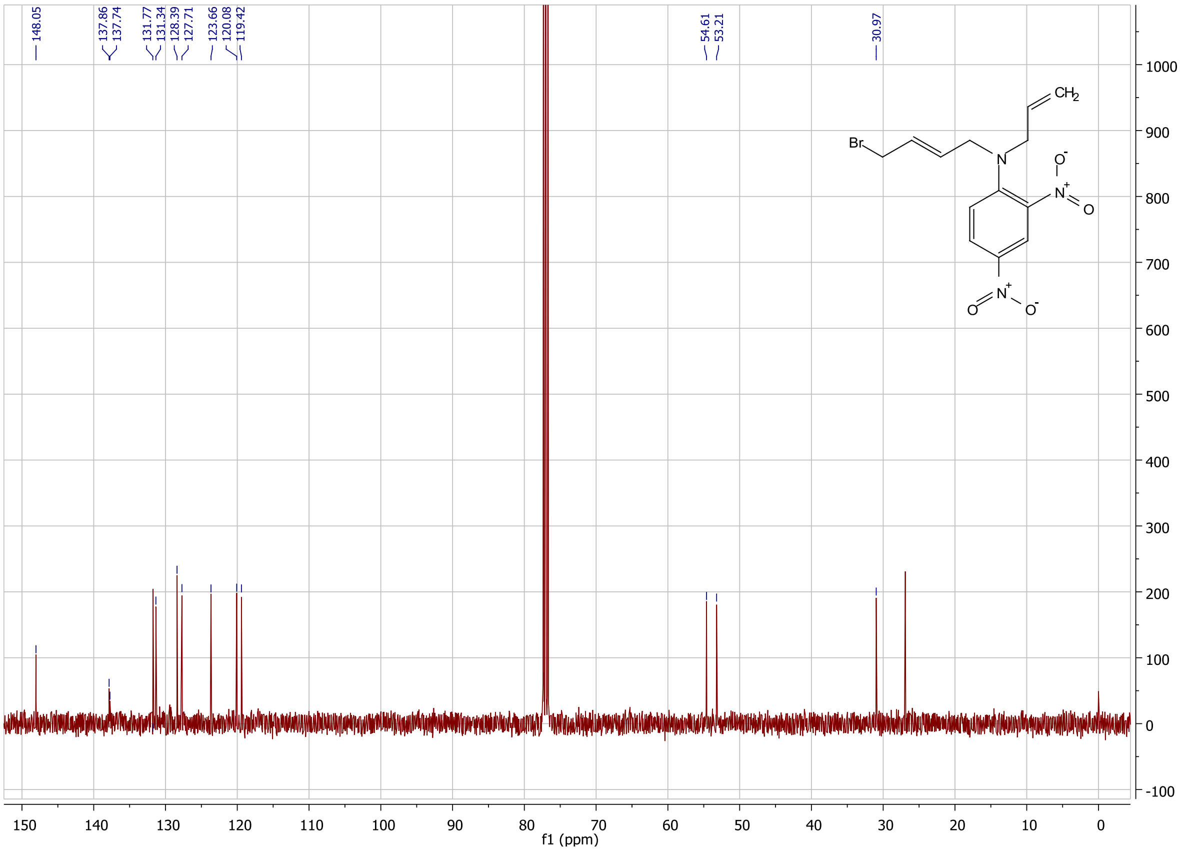

148.05
137.86
137.74
131.77
131.34
128.39
127.71
123.66
120.08
119.42
54.61
53.21
30.97
CH2
Br
N
O-
N+
O
N+
O
O-
f1 (ppm)

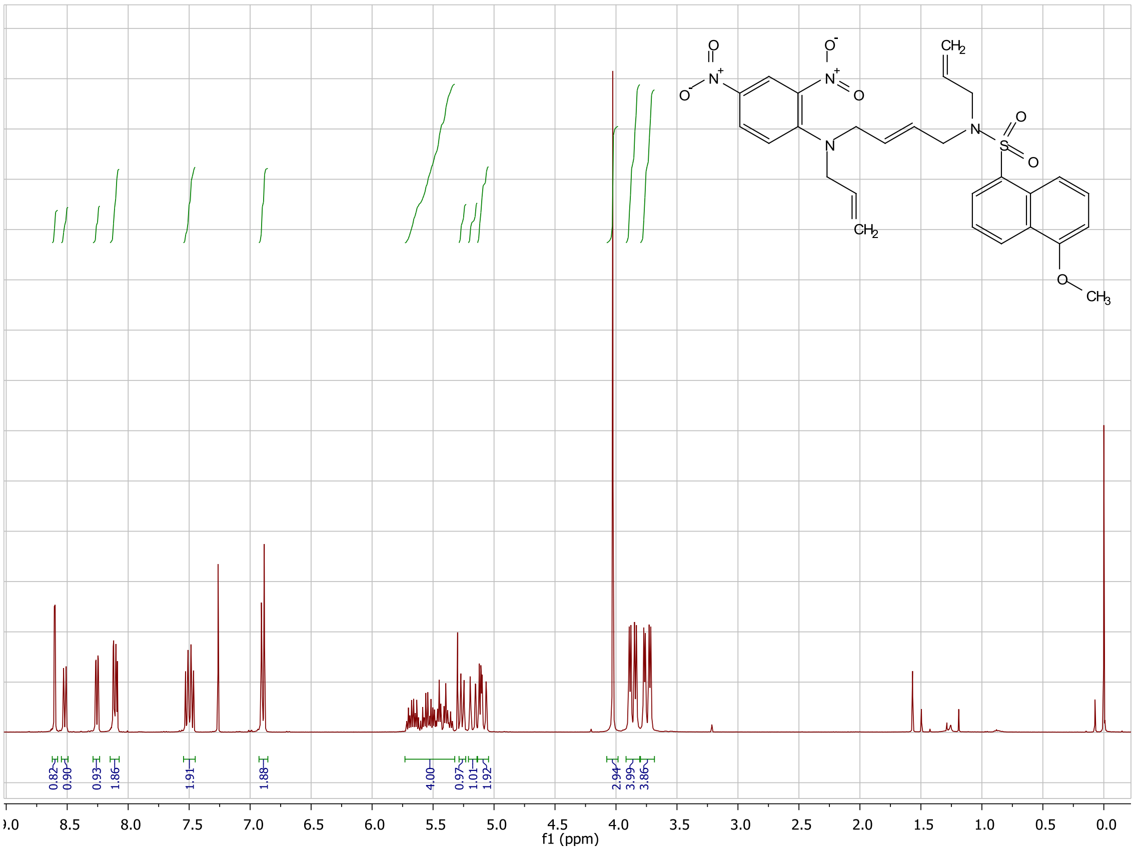
0.82
0.90
0.93
1.86
1.91
1.88
4.00
0.97
1.01
1.92
2.94
3.99
3.86
f1 (ppm)

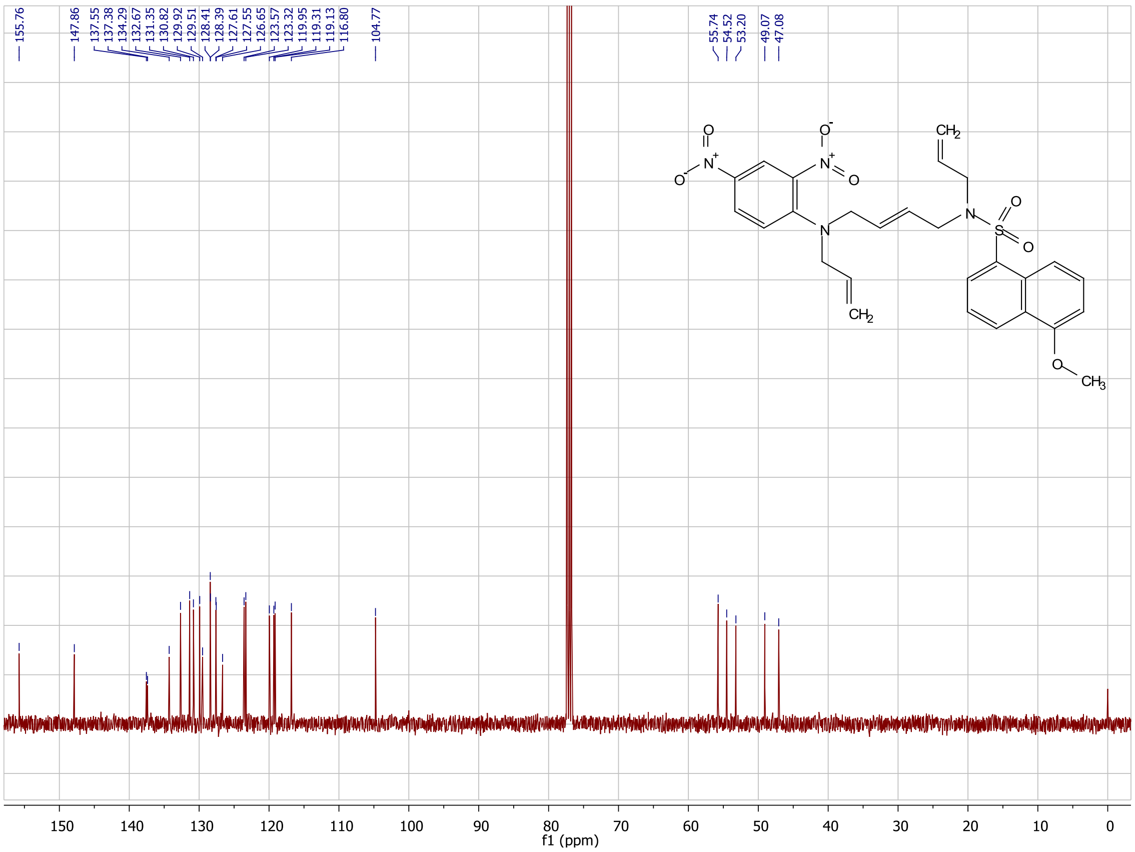
155.76
147.86
137.55
137.38
134.29
132.67
131.35
130.82
129.92
129.51
128.41
128.39
127.61
127.55
126.65
123.57
123.32
119.95
119.31
119.13
116.80
104.77
55.74
54.52
53.20
49.07
47.08
f1 (ppm)
